# *Mesosphaerum suaveolens* Essential Oil Attenuates Inflammatory Response and Oxidative Stress in LPS-Stimulated RAW 264.7 Macrophages by Regulating NF-κB Signaling Pathway

**DOI:** 10.3390/molecules28155817

**Published:** 2023-08-02

**Authors:** Omprakash Mohanta, Asit Ray, Sudipta Jena, Ambika Sahoo, Soumya Swarup Panda, Prabhat Kumar Das, Sanghamitra Nayak, Pratap Chandra Panda

**Affiliations:** Centre for Biotechnology, Siksha ‘O’ Anusandhan (Deemed to be University), Kalinga Nagar, Bhubaneswar 751003, India

**Keywords:** anti-inflammatory, essential oil, lipopolysaccharide, RAW 264.7 cells, *Mesosphaerum suaveolens*, NF-κB pathway

## Abstract

*Mesosphaerum suaveolens* (L.) Kuntze (Syn. *Hyptis suaveolens* (L.) Poit.) is a wild essential-oil-bearing plant having multiple uses in traditional medicine, perfumery, food, agriculture, and pharmaceutical industries. The present paper is the first report on the in vitro anti-inflammatory effects of the leaf essential oil of *M. suaveolens* (MSLEO) and unravels its molecular mechanism in LPS-stimulated RAW 264.7 macrophage cells. GC-MS analysis of the essential oil (EO) isolated from the leaves by hydro-distillation led to the identification of 48 constituents, accounting for 90.55% of the total oil, and β-caryophyllene (16.17%), phyllocladene (11.85%), abietatriene (11.46%), and spathulenol (7.89%) were found to be the major components. MSLEO treatment had no effect on the viability of RAW 264.7 cells up to a concentration of 100 μg/mL, and the EO was responsible for a reduction in proinflammatory cytokines like IL-6, IL-1β, and TNF-α, a decrease in intracellular ROS production, and the restoration of oxidative damage by elevating the levels of endogenous antioxidative enzymes like CAT, SOD, GPx, and GSH. RT-qPCR analysis indicated that MSLEO reduced the mRNA expression levels of iNOS and COX-2 as compared to the LPS-induced group. In addition, a confocal microscopy analysis showed that MSLEO inhibited the translocation of NF-κB from the cytosol to the nucleus. The results of this experiment demonstrate that MSLEO possesses significant anti-inflammatory potential by preventing the activation of NF-κB, which, in turn, inhibits the downstream expression of other inflammatory mediators associated with the activation of the NF-κB pathway in LPS-induced RAW 264.7 cells. Thus, the leaf essential oil of *M. suaveolens* may prove to be a promising therapeutic agent for the treatment of inflammation, and targeting the NF-κB signaling pathway may be considered as an attractive approach for anti-inflammatory therapies.

## 1. Introduction

Inflammation is a biological response triggered by damage to living tissues by pathogenic bacteria, viruses, fungi and protozoa, toxic chemicals, and injury, and through this mechanism, the injurious stimuli are removed, thereby facilitating the initiation of the healing process [1,2]. Under normal physiological conditions, it acts like a mitigation process and contributes to restoring tissue homeostasis and resolving acute inflammation. However, uncontrolled acute inflammation may become chronic over a period of time and lead to disease conditions like rheumatism and arthritis [3]; atherosclerosis [4]; type-2 diabetes; cardiovascular, pulmonary, and autoimmune diseases [5]; gastrointestinal disease [6]; metabolic syndromes [7]; and cancer [5].

Macrophages play a significant role in the human immune system. Under chronic inflammatory conditions, macrophages are over-activated, resulting in oxidative stress and the enhanced production of inflammatory mediators, such as reactive oxygen species (ROS), nitric oxide (NO), prostaglandin E2 (PGE2), Interleukin-6 (IL-6), Interleukin-1β (IL-1β), and tumor necrosis factor alpha (TNF-α), thereby contributing to tissue damage and consequent functional impairment [8]. Macrophages are activated by lipopolysaccharides (LPS), a bacterial endotoxin that activates cells of the innate immune system, which synthesize proinflammatory mediators and cytokines through several pathways, of which NF-κB (nuclear factor of κ-light chain of enhancer-activated B cells) and mitogen-activated protein kinase (MAPK) signaling pathways are noteworthy [9]. In addition, an elevated level of ROS production is associated with increased oxidative stress, which negatively impacts cellular structures like membranes, lipids, proteins, lipoproteins, and deoxyribonucleic acid, leading to disease conditions such as inflammatory disorders, aging, degenerative nerve diseases, and cancer [10,11]. When activated, NF-κB has a pivotal role to play in controlling the expression of several inflammatory genes and the enhanced synthesis of proinflammatory mediators, resulting in the regulation of cell growth, proliferation, and apoptosis [12]. In view of this, the mechanism of NF-κB signaling pathway regulation has been considered as an efficient therapeutic strategy to deal with inflammation. The suppression of dysregulated inflammatory and oxidative stress is essential to mitigate the risk associated with inflammatory conditions, which necessitates the development of anti-inflammatory agents [13].

Non-steroidal anti-inflammatory drugs (NSAIDs) are a group of therapeutic drugs widely used to reduce pain, decrease inflammation and fever, and prevent blood clots. They act on the human body by inhibiting the cyclooxygenase (COX) enzyme, which is required for the bioconversion of arachidonic acid to inflammatory prostaglandins (PGs). Because of their anti-inflammatory, anti-pyretic, and pain-relieving properties, NSAIDs have become the drugs of choice for treating several inflammatory conditions, like rheumatism, arthritis, and body pain [14]. However, recent studies indicate that the long-term use of these drugs may lead to gastrointestinal, cardiovascular, hepatic, renal, cerebral, and pulmonary complications [15,16]. Because of the well-known side effects of NSAIDs, the world’s attention has now been diverted toward natural compounds, such as dietary supplements and plant products, which have long been used to relieve muscle and joint pain and symptoms of inflammation-related diseases [17]. Most of these natural compounds behave like anti-inflammatory drugs by inhibiting COX and NF-κB inflammatory pathways in a manner quite similar to that of NSAIDs [18].

Owing to their therapeutic potential and relatively few or no adverse effects, anti-inflammatory compounds of plant origin have assumed greater significance and have become becoming increasingly popular in recent times for the treatment of a wide range of inflammatory disorders [19]. Among plant-derived natural agents, essential oils (EOs) are known to have antioxidant and anti-inflammatory activities through free radical scavenging, the enhancement of the capacity of the antioxidant defense mechanism, and the downregulation of inflammatory mediators [20,21]. The anti-inflammatory properties of plant-derived essential oils have been established under in vitro conditions by several researchers in the past using different designated cell lines [22,23]. According to Miguel [20], the terpenoids (monoterpenoids and sesquiterpenoids) in essential oils suppress the level of the expression of genes responsible for the production of inflammatory mediators in in vitro anti-inflammatory studies. 

The genus *Mesosphaerum* P. Br. (Lamiaceae) is represented by 24 accepted species distributed in tropical and sub-tropical America [24]. Of these, *Mesosphaerum suaveolens* (L.) Kuntze (Syn. *Hyptis suaveolens* (L.) Poit.), commonly known as Bush mint or Pignut, is native to Mexico and tropical America. However, it has been established as an aggressive invasive weed in the tropics, especially in wet and warm areas [25,26]. In India, this potent invader is naturalized in almost all types of habitats, from wastelands to agricultural fields and forests. The leaves, stems, roots, and seeds of this species are widely used in local and folk medicines against respiratory and gastrointestinal diseases, indigestion, dysentery, boils, wounds, tumors, headache, cold, fever, cramps, body pain, skin diseases, cancer, leukorrhea, renal disorders, and inflammatory conditions [27,28]. The plant is reported to possess significant antioxidant, anti-inflammatory, wound healing, cytotoxic, anti-malarial, anti-diabetic, and antimicrobial properties. *M. suaveolens* is used as an antidote for snake bites [29], as a botanical insecticide [30], and also as a mosquito and insect repellant [31]. The glutinous seeds of the plant, known as “Chan” make a good food with potent nutraceutical potential and are used in traditional medicine in several countries [32,33]. 

The yield and composition of the essential oil of *M. suaveolens* have been studied by many researchers in the past from different parts of the world, and the related literature has been reviewed [34,35]. Considerably wide variability in the yield and chemical composition of the major phytoconstituents of essential oils has been reported, which could be attributed to the geographical origin, season, soil, and climatic conditions [36,37]. The essential oil of the species possesses potential antimicrobial, insecticidal, insect-repellent, and larvicidal activities [35]. In addition, it has reported antioxidant [38,39], cytotoxic [40], and anti-cancer [39,41] properties. 

Though there is some published literature on the anti-inflammatory effects of the ethanolic and methanolic extracts of *M. suaveolens* [42,43,44], to date, there is no published work pertaining to the anti-inflammatory properties of its essential oil or the underlying molecular mechanism of its action. For the first time, the present paper reports the in vitro anti-inflammatory activities of the leaf essential oil of *Mesosphaerum suaveolens* (MSLEO) and unravels the molecular mechanism of its action in LPS-stimulated RAW 264.7 cells. 

## 2. Results and Discussion

### 2.1. Chemical Composition of M. suaveolens Leaf Essential Oil (MSLEO)

MSLEO was pale-yellow in color and mint-like in smell, with a yield of 0.23% on a dry weight basis. The average oil yield was found to be higher compared to those determined for the same species by Mallavarapu et al. [45], Peerzada [46], and Bezerra et al. [40] but lower than those recorded by Malele et al. [47], Lohani et al. [48], and Xu et al. [39]. GC-MS analysis of the essential oil revealed that the oil is composed of 48 compounds, which account for 90.55% of the total essential oil (EO) (Table 1, Figure S1). The major constituents of MSLEO were β-caryophyllene (16.17%), phyllocladene (11.85%), abietatriene (11.46%), spathulenol (7.89%), 8,13-abietadien-18-ol (6.65%), and abietadiene (3.79%). The major chemical classes of compounds in the EO were diterpene hydrocarbons (28.82%), sesquiterpene hydrocarbons (25.74%), sesquiterpene alcohols (13.76%), and oxygenated diterpenes (13.34%). β-Caryophyllene has been detected to be the predominant component of essential oils of *M. suaveolens* by several researchers in the past [39,40,47,49]. This natural bicyclic sesquiterpene, present in many edible plants and essential oils, is known to be an effective anti-inflammatory agent [50,51,52,53]. By inhibiting the production of inflammatory mediators and regulating cell proliferation, β-caryophyllene exerts anti-inflammatory effects [54,55]. This led to the belief that β-caryophyllene or plants rich in β-caryophyllene have anti-inflammatory actions and may serve as new sources of drugs for treating viral infection, improving immunity, and reducing inflammation [56].

Among other major constituents of MSLEO, spathulenol is also known to exhibit anti-inflammatory effects, according to several researchers [58,59]. The essential oil of *Psidium guineense,* with spathulenol as the major constituent, exhibited strong anti-inflammatory activity against formalin-induced paw edema and carrageenan-induced mechanical hyperalgesia and paw edema in male Swiss mice [60]. Abietadiene and abietatriene, a group of aromatic abietanes, detected as dominant constituents of MSLEO, are also known to be anti-inflammatory in nature [61,62]. In addition, several minor constituents, like eucalyptol, β-selinene, caryophyllene oxide, and phytol, present in MSLEO have been reported to have anti-inflammatory activities [63,64,65]. The anti-inflammatory activity of MSLEO could be due to the predominance of β-caryophyllene, spathulenol, etc., or because of the synergistic effect of one or more major and minor phytoconstituents of the oil.

As can be seen from Table 1, MSLEO is mainly composed of terpenes and terpenoids, which are known to inhibit the NF-κB signaling pathway through IκB phosphorylation, DNA binding, p65 translocation, etc. [66,67,68,69]. These phytochemicals inhibit phosphorylation and IκB protein degradation, and subsequently, the active IκB-free NF-κB is translocated from the cytoplasm to the nucleus, leading to the transcription of proinflammatory cytokines [70]. β-Caryophyllene, a dominant constituent of MSLEO, is reported to act as an anti-inflammatory agent through the suppression of the main mediators of inflammation [52,55]. The expression of NF-ƙB is known to be downregulated by β-caryophyllene, thereby diminishing inflammatory and apoptotic responses [71,72,73,74]. Other major diterpenoid compounds, like phyllocladane, phytol, and abietanes, that were detected in MSLEO are reported to inhibit NF-κB activity by reducing the levels of cytokines and oxidative stress [63,75,76]. The anti-inflammatory effect of spathulenol has also been established through a mouse model of carrageenan-induced paw edema and pleurisy. Similarly, another minor constituent, sabinene, also showed anti-inflammatory activity by substantially regulating the production of NO and proinflammatory cytokines [77,78].

### 2.2. MSLEO Had No Impact on the Viability and Morphology of RAW 264.7 Cells 

**Figure 1:**
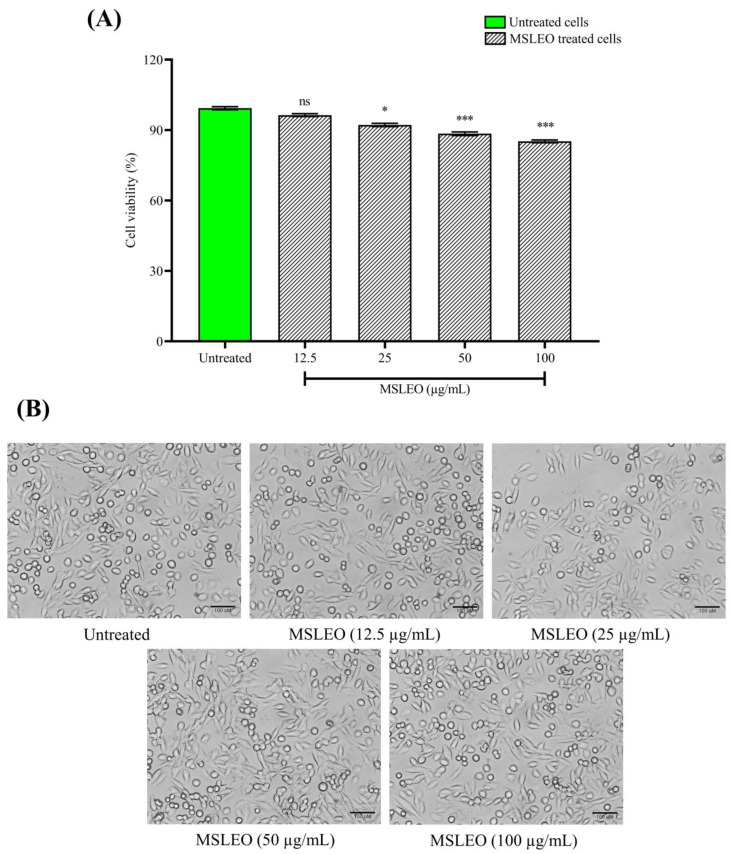
Cytotoxicity and morphological alteration in RAW 264.7 cells after treatment with different concentrations of MSLEO. (**A**) Cell viability as measured by MTT assay by treating cells with different concentrations of MSLEO (12.5–100 µg/mL) and incubating them for 24 h. (**B**) Morphological alterations in cells visualized under an inverted microscope after treatment with various concentrations of MSLEO (scale bar = 100 µm). Each value indicates the mean ± SD of 3 independent experiments. Statistical significance was measured by one-way ANOVA followed by the Tukey test. ns > 0.5, * *p* < 0.05, *** *p* < 0.001 between the untreated and MSLEO-treated cells.

The MTT (3-[4,5-dimethylthiazol-2-yl]-2,5 diphenyl tetrazolium bromide) assay was performed to test the cytotoxicity of MSLEO. RAW 264.7 cells were treated with different doses of MSLEO between 12.5 µg/mL and 100 µg/mL (i.e., 12.5, 25, 50, and 100 µg/mL) to determine the level of toxicity and fix the non-toxic range of concentration to undertake anti-inflammatory assays. The half-maximal inhibitory concentration (IC_50_) of MSLEO was determined to be 324.17 µg/mL, as shown in Figure 1A. The results of the assay showed that MSLEO, up to a dose of 100 µg/mL, did not affect cell viability, which remained greater than 85%. Further, microscopic observation revealed that MSLEO, up to a 100 µg/mL concentration, did not show much cell spreading or pseudopodia formation, indicating that most of the cells were viable (Figure 1B). The results established that MSLEO did not adversely impact the viability and morphology of RAW 264.7 cells, and this could be due to the anti-inflammatory properties of MSLEO.

Based on these data, MSLEO at a lower concentration range (12.5 µg/mL) and a higher concentration range (100 µg/mL) were considered for the subsequent studies relating to inflammation and associated responses. Jena et al. [19] also observed no toxicity up to a 100 µg/mL concentration of the essential oil of *Neocinnamomum caudatum*. Similarly, treatment with 50 μg/mL *Neolitsea sericea* essential oil also maintained cell viability at 85% [79]. Using the MTT assay, Raka et al. [2] found that the viability of cells was not affected at all and remained greater than 90% with treatment with the Pingyin rose (*Rosa rugosa*) essential oil up to 100 µg/mL. In general, it is essential to know the optimal concentration of an essential oil before it is used as an anti-inflammatory agent [80]. 

### 2.3. MSLEO Treatment Attenuates LPS-Induced Increase in Apoptosis 

Apoptosis is the genetically regulated death of cells to ensure homeostasis and can be activated by two main pathways, i.e., death receptors and mitochondrial pathways. The essential oils of a number of plant species are reported to induce apoptosis marked by cytomorphological alterations like cell shrinkage, chromatin condensation, fragmentation of DNA accompanied by mitochondrial dysfunction, protein cleavage, etc. [81]. An abnormality in apoptosis may lead to several types of illnesses, including autoimmune diseases, neurodegenerative disorders, bacterial and viral infections, cardiac problems, and cancer [82]. An enhanced level of ROS generation, activation of protein kinase B, phosphorylated MAPKs, mitochondrial stress, and caspase activation are the main mechanisms of action by which plant essential oils induce programmed cell death [81]. 

**Figure 2:**
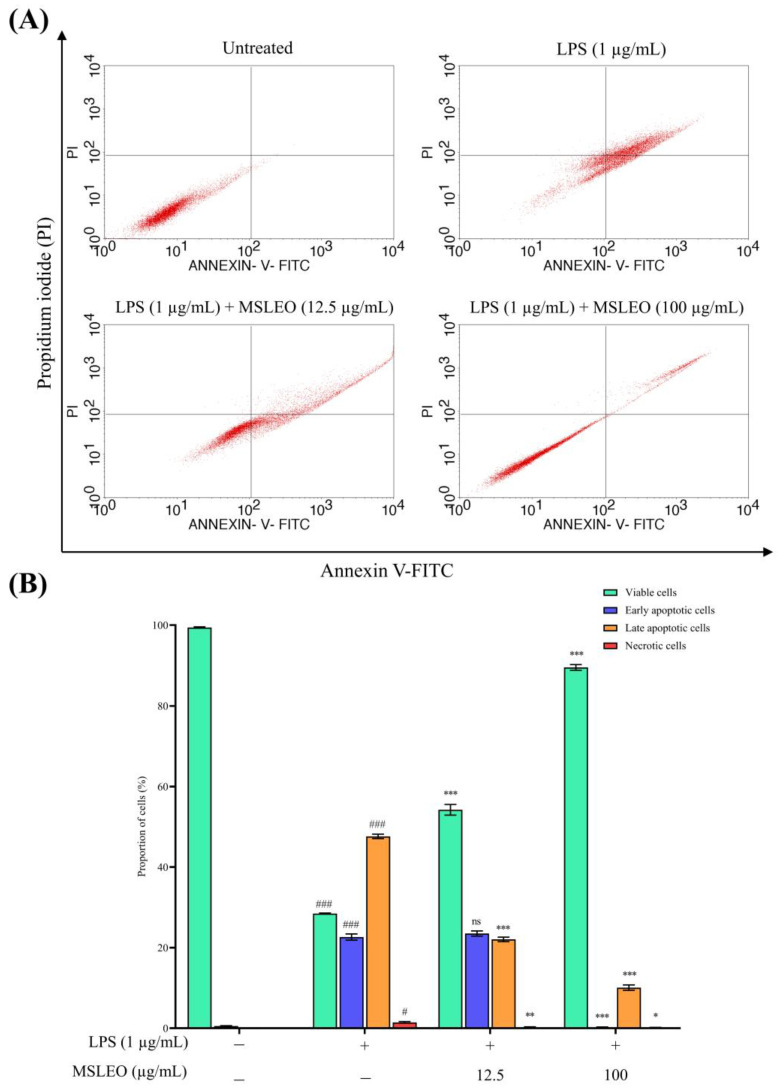
Effect of MSLEO on LPS-induced apoptosis. RAW 264.7 cells were pretreated with LPS (1 µg/mL), followed by different doses of MSLEO (12.5 and 100 µg/mL) for 24 h, stained with FITC-conjugated Annexin V and PI, and analyzed by flow cytometry. (**A**) Flow cytometry dot plots showing apoptosis of RAW 264.7 cells in different treatment groups (**B**). The proportion of viable cells, necrotic cells, and apoptotic cells (including early and late apoptotic cells) in different treatment groups determined by analyzing 10,000 gated cells using the flow cytometric method. Each value indicates the mean ± SD of three independent experiments. Statistical significance was determined by one-way ANOVA followed by the Tukey test. ^#^ *p* < 0.05, ^###^ *p* < 0.001 between the untreated and LPS-treated (1 µg/mL) cells; ns > 0.5, * *p* < 0.05, ** *p* < 0.01, *** *p* < 0.001 between the LPS (1 µg/mL) and MSLEO-treated cells.

In the present experiment, the externalization of phosphatidyl serine (PS) and cell membrane integrity was assessed by Annexin V–fluorescein isothiocyanate (FITC) and propidium iodide (PI) staining using a flow cytometer to study the effects of MSLEO on LPS-induced macrophage cells. When macrophage cells were activated with 1 µg/mL LPS for 24 h, the percentage of late (Annexin V^+ve^/PI^+ve^) apoptotic cells was significantly enhanced from 0.43% to 70.05%, and cell viability (Annexin V^−ve^/PI^−ve^) was drastically reduced from 99.57% to 28.61%, as depicted in Figure 2. Further, when these LPS-stimulated cells were co-cultured with a concentration of 12.5 µg/mL MSLEO, the proportion of apoptotic cells decreased from 71.39% to 45.09%, and the population of viable cells was nearly doubled from 28.61% to 54.73% in comparison to those treated with LPS only. 

However, the treatment of murine macrophage cells with a dose of 100 µg/mL MSLEO drastically brought down the percentage of apoptotic cells from 70.05% to 10.14% and elevated the number of viable cells by more than 3 times (i.e., from 28.61% to 89.45%). Additionally, the proportion of necrotic cells decreased from 1.34% to 0.18% when LPS-induced macrophage cells were incubated with different concentrations of MSLEO. As reported earlier, the LPS model induces cell death by apoptosis through the autocrine secretion of TNF-α at the early stage and by the production of NO at the late apoptotic stage [83,84]. The data obtained from the Annexin V-FITC and PI double-staining assay suggested that MSLEO can attenuate the LPS-induced increase in apoptosis in a dose-dependent manner, consistent with the cytoprotective and anti-inflammatory activities of MSLEO.

### 2.4. MSLEO Inhibits the LPS-Induced Increase in Expression of Proinflammatory Cytokines 

One of the pathways through which plant essential oils primarily inhibit or attenuate inflammation is by downregulating the expression of the mRNA or protein of three key proinflammatory cytokines: IL-1β, IL-6, and TNF-α [85]. These cytokines regulate the immune response to inflammation through a complex network of interactions. They are stimulated in response to the invasion of the host by pathogenic microorganisms and are responsible for the recruitment and amplification of immune cells [86]. 

**Figure 3:**
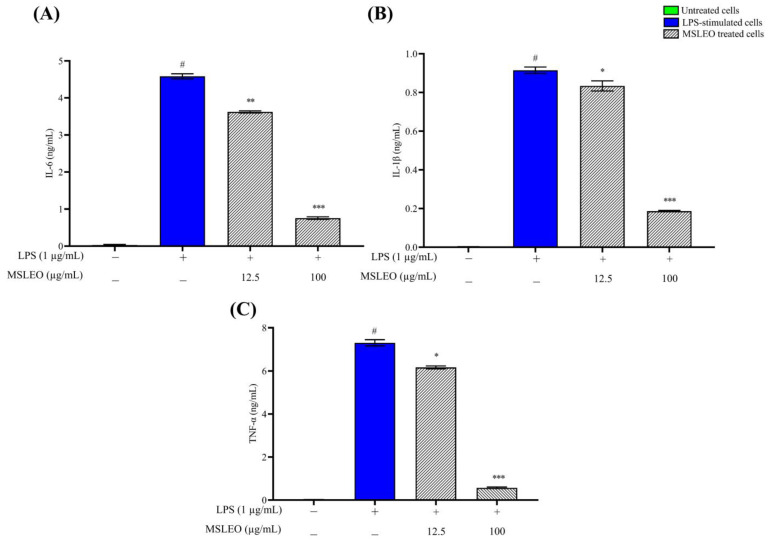
Effect of MSLEO on production of proinflammatory cytokines. The cells were pretreated with LPS (1 µg/mL) and then incubated with different dosages (12.5 and 100 µg/mL) of MSLEO for 24 h. The expression patterns of (**A**) IL-6, (**B**) IL-1β, and (**C**) TNF-α were determined using ELISA. Each value indicates the mean ± SD of three independent experiments. Statistical significance was calculated by one-way ANOVA followed by the Tukey test. ^#^ *p* < 0.001 between the untreated and LPS-treated (1 µg/mL) cells; * *p* < 0.05, ** *p* < 0.01, *** *p* < 0.001 between the LPS (1 µg/mL) and MSLEO-treated cells.

Being an endotoxin, LPS can activate macrophages and stimulate the release of several proinflammatory cytokines, which are involved in the upregulation of inflammatory reactions [87]. In the present investigation, the levels of expression of the above proinflammatory cytokines were first assessed in LPS-stimulated cells. Thereafter, the effect of MSLEO on the production of cytokines was measured by an ELISA (enzyme-linked immunosorbent assay) kit using a microplate reader. As depicted in Figure 3, after 24 h of exposure to LPS (1 µg/mL), significant increases in the expression levels of IL-6, IL-1β, and TNF-α were noticed as compared to the untreated group. Interestingly, incubation of these pre-activated macrophage cells treated with MSLEO at 12.5 and 100 µg/mL for 24 h was able to effectively reduce the levels of expression of these main proinflammatory cytokines in a dose-dependent manner. For instance, the administration of MSLEO at a 100 µg/mL concentration could reduce the levels of IL-6, IL-1β, and TNF-α by 6.1-fold, 4.8-fold, and 13.5-fold, respectively, compared to the LPS-treated groups.

The results of the present study are in agreement with the findings of earlier studies, where essential oils of *Cinnamomum* and *Neocinnamomum* species were found to suppress the production of the above proinflammatory cytokines generated by LPS depending on the concentration used [19,88,89]. The essential oils extracted from many other plant species, such as *Citrus* species, *Artemisia fukudo*, *Chimonanthus praecox*, *Myrciaria tenella*, *Eucalyptus camaldulensis*, *Verbesina macrophylla*, *Lavandula angustifolia*, *Melaleuca alternifolia*, and *Cymbopogon martinii*, have been reported to suppress the transcription of IL-6, IL-1β, and TNF-α by limiting their mRNA expression [12].

β-Caryophyllene, a major constituent of MSLEO, is known to exert strong anti-inflammatory effects by inhibiting the synthesis of proinflammatory mediators [90]. It is reported to inhibit the transcription of the above mediators in central nervous system immune effector cells (C6) treated at a dose of 10 mg/kg [50,91]. Further, the above data suggest that β-caryophyllene protects neurons from ischemia-induced cell death and acts as an anti-inflammatory agent. In addition, spathulenol and caryophyllene oxide in the essential oils have an important role in the process of the inhibition of these inflammatory mediators [92]. The anti-inflammatory activity of MSLEO through the suppression of the expression of different proinflammatory cytokines may be attributed to the dominance of β-caryophyllene, spathulenol, and other constituents and the synergistic effect of some major and minor constituents.

### 2.5. MSLEO Downregulates LPS-Induced Increase in iNOS and COX-2 mRNA Expression

**Figure 4:**
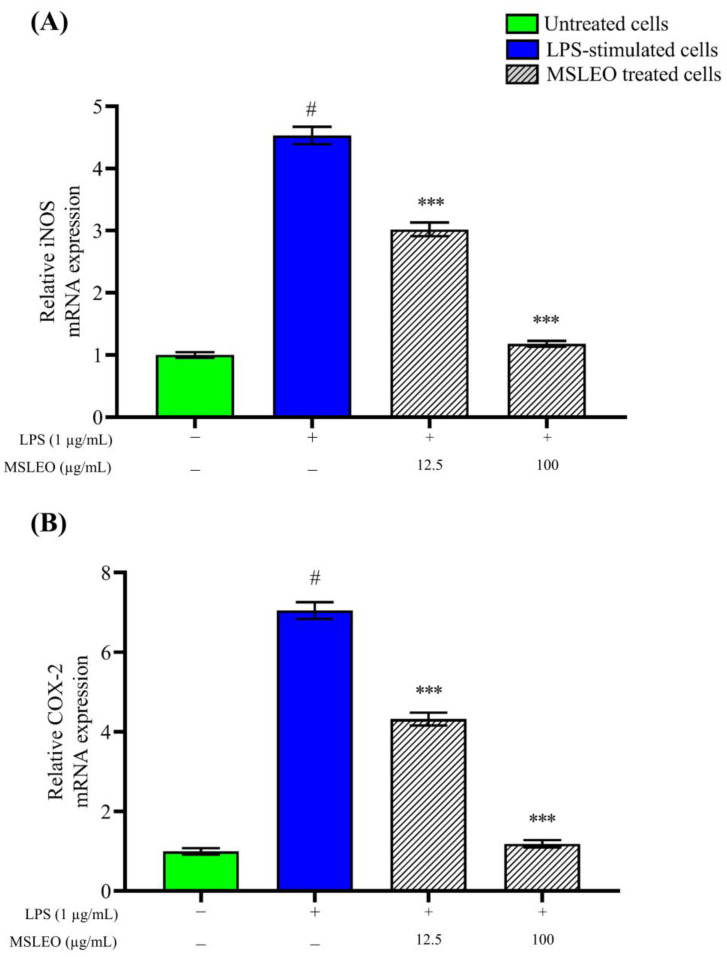
Effect of MSLEO on iNOS and COX-2 expression. RAW 264.7 cells were stimulated with LPS (1 µg/mL) for 2 h, and then they were treated with MSLEO at 12.5 µg/mL and 100 µg/mL concentrations for 24 h. The relative mRNA expression of (**A**) iNOS and (**B**) COX-2 determined by RT-qPCR analysis. Each value indicates the mean ± SD of three independent experiments. Statistical significance was calculated by one-way ANOVA followed by the Tukey test. ^#^ *p* < 0.001 between the untreated cells and LPS-treated (1 µg/mL) cells; *** *p* < 0.001 between the LPS (1 µg/mL) and MSLEO-treated cells.

In order to evaluate the anti-inflammatory potential of MSLEO, the levels of iNOS and COX-2 mRNA expression were estimated by real-time quantitative PCR (RT-qPCR). RAW 264.7 cells were treated with different concentrations of MSLEO (12.5 and 25 µg/mL) and with LPS and incubated for 24 h. The mRNA expression levels of iNOS and COX-2 were determined after incubation. As shown in Figure 4, the expression levels of iNOS and COX-2 increased many-fold upon incubation with 1 µg/mL LPS. When these pre-LPS-stimulated macrophages were treated with the minimum and maximum non-toxic doses of MSLEO (i.e., 12.5 and 100 µg/mL, respectively), the over-expression of iNOS and COX-2 mRNA was downregulated in a dose-dependent manner (Figure 4A,B). Interestingly, with a higher dose of MSLEO, a significant reduction in the expression of iNOS and COX-2 mRNA was observed as compared to the LPS-treated group, thereby restoring homeostasis. 

iNOS, the most common isoform of NO, is produced when cells are stimulated with inflammatory cytokines and LPS [93,94]. Being a potent activator of iNOS, LPS interacts with Toll-like receptor 4 (TLR4) to activate macrophages [95]. Further, the role of the COX-2 enzyme is crucial in the process of the synthesis of PGE2, which is essentially an inflammatory mediator. As per Wei et al. [86], the production of PGE2 and NO is a closely linked process. In the mitigation of inflammatory conditions, the reduction in the levels of iNOS and COX-2 expression is considered crucial. One of the pathways by which plant essential oils primarily attenuate inflammatory responses is by inhibiting inducible nitric oxide synthase [96]. In addition, essential oils interfere with the arachidonic acid metabolic pathway and inhibit COX and LOX (Lipoxygenase) activity with a consequent reduction in PGE2, thus attenuating the deteriorating inflammatory condition [12]. Since NF-κB, in the activated state, facilitates the increased production of inflammatory cytokines, chemokines, and mediator enzymes [97], the regulation of the NF-κB signaling pathway has been widely used as a therapeutic route in the treatment of inflammatory conditions.

Kim et al. [98] found that the essential oil of *Mentha arvensis* exhibits anti-inflammatory activity by inhibiting proinflammatory cytokines and mediators through the suppression of COX-2 and iNOS expression. Similarly, the expression of the above two enzymes was found to be inhibited by essential oils of *Chamaecyparis obtusa*, *Perilla frutescens*, and *Origanum vulgare* [99,100]. The results of RT-qPCR and the concentration-dependent reduction in iNOS and COX-2 mRNA expression after treatment with MSLEO suggest that the oil has potent anti-inflammatory effects that can inhibit certain elements of the NF-κB pathway and ultimately minimize inflammatory responses. 

### 2.6. MSLEO Treatment Decreased LPS-Induced Increase in ROS Levels 

Oxidative stress is reported to be associated with the pathogenesis of several metabolic and chronic disorders, including inflammatory conditions like cancer and cardiovascular disorders [101,102]. There is an increasing amount of evidence that ROS play a crucial role in the occurrence, progression, and resolution of the inflammatory response [103]. In addition, ROS act as signaling molecules for the regulation of cell survival, cell death, differentiation, cell signaling, and inflammation-related factor production [104]. However, prolonged or over-production of ROS by polymorphonuclear neutrophils (PMNs) at the inflammation site is responsible for endothelial injury, causing chronic inflammation underlying many neurodegenerative, cardiovascular, and metabolic diseases [105]. The majority of ROS are produced by aerobic cells during the mitochondrial respiratory chain and endogenous metabolic reactions [106,107], which are required to activate a variety of signaling pathways in both plants and animals. However, during LPS stimulation, RAW 264.7 cells produce excessive amounts of ROS/free radicals [108], and the endogenous antioxidants are inadequate to handle this condition, resulting in oxidative stress. 

**Figure 5:**
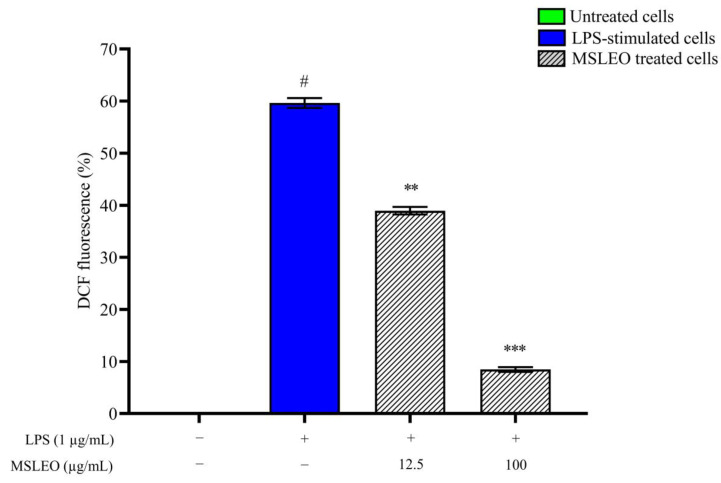
Effect of MSLEO on intracellular ROS levels in LPS-stimulated RAW 264.7 cells. The pre-LPS-stimulated (1 µg/mL) murine macrophages were incubated with 12.5 µg/mL and 100 µg/mL MSLEO for 24 h, followed by treatment with the DCFH-DA (10 µM) fluorescent probe for 30 min and analysis by flow cytometry. Representative bar graphs show the mean DCFH-DA fluorescence intensity of different treatment groups. Each value indicates the mean ± SD of three independent experiments. Statistical significance was calculated by one-way ANOVA followed by the Tukey test. ^#^ *p* < 0.001 between the untreated and LPS-treated (1 µg/mL) cells; ** *p* < 0.01, and *** *p* < 0.001 between the standard LPS (1 µg/mL) and MSLEO-treated cells.

In the present study, the H_2_DCF-DA (2′,7′-dichlorodihydrofluorescein diacetate) cellular ROS assay kit was utilized to evaluate the effect of MSLEO on the intracellular ROS generation causing oxidative stress. Intracellular ROS oxidized non-fluorescent H_2_DCF-DA into fluorescent DCF (dichlorodihydrofluorescein), and the resulting fluorescence intensity was analyzed by a flow cytometer and correlated with the quantification of ROS produced during inflammation. As outlined in Figure 5, after 24 h of exposure to LPS (1 μg/mL), the percentage of RAW 264.7 cells emitting DCF fluorescence increased from 0.08% to 59.68%, indicating an escalated intracellular ROS level in LPS-treated cells in comparison to untreated cells. Meanwhile, intracellular ROS production in LPS-treated macrophages was substantially reduced upon the administration of 12.5 and 100 µg/mL MSLEO for 24 h. MSLEO at 12.5 µg/mL was able to suppress the intracellular ROS level by 1.5-fold, but at a higher concentration (100 µg/mL), the extent of ROS suppression was 7.1-fold in comparison with groups treated with LPS alone. The findings of this study are in agreement with the results obtained by earlier researchers, who found that essential oils of plant origin effectively neutralize intracellular ROS [109,110]. The reduction in NF-κB activity and the enhancement of mitochondrial membrane potential are indications of a reduction in the levels of ROS after treatment with MSLEO.

Inflammation and oxidative stress are pathophysiological events that are intricately related to each other [111,112]. In many chronic diseases, the co-existence of inflammation and oxidative stress has been reported. While cells liberate several reactive species at the site of inflammation, resulting in oxidative stress, many ROS/RNS can influence intracellular signaling cascades leading to the enhanced expression of proinflammatory genes [113,114]. The removal of pathogenic organisms from the body is the main purpose of inflammation, and for this, ROS production by phagocytes is important. While normal levels of ROS are important to reach homeostasis, normal levels of ROS are also equally important for killing pathogens. The uncontrolled production of ROS by different antioxidant mechanisms may lead to tissue damage. The antioxidant mechanism of the cell has a pivotal role to play in maintaining this intricate balance [112].

### 2.7. MSLEO Enhances Endogenous Antioxidant Enzyme Activities 

Endogenous antioxidant enzymes, like glutathione peroxidase (GPx), catalase (CAT), and superoxide dismutase (SOD), counteract the pro-oxidant effects of ROS and thereby prevent damage to cells and tissues [103,115]. These antioxidant enzymes sequentially remove redundant ROS and prevent oxidative bursts. SOD initially catalyzes harmful superoxide radicals into molecular oxygen (O_2_) and hydrogen peroxide (H_2_O_2_). Subsequently, CAT or GPx converts H_2_O_2_ into the innocuous end-product, water [116]. 

**Figure 6:**
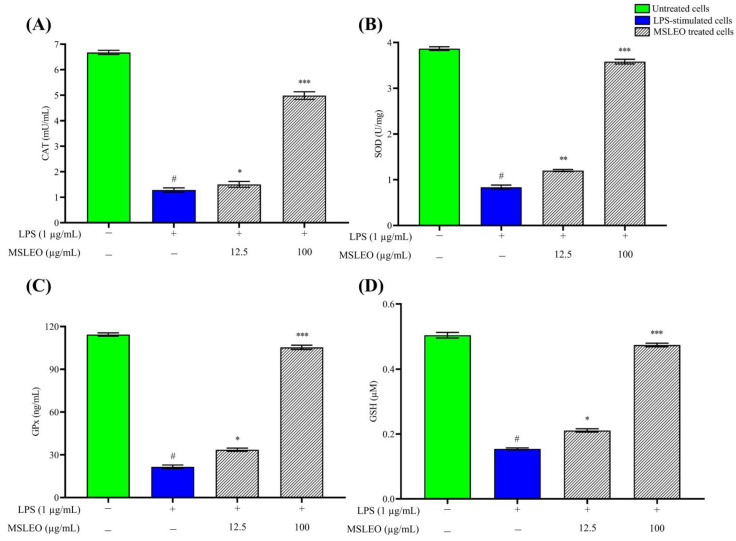
Effect of various concentrations (12.5–100 µg/mL) of MSLEO on the activity of endogenous antioxidant enzymes: (**A**) catalase (CAT); (**B**) superoxide dismutase (SOD); (**C**) glutathione peroxidase (GPx); and (**D**) glutathione (GSH). Each value indicates the mean ± SD of three independent experiments. Statistical significance was calculated by one-way ANOVA followed by the Tukey test. ^#^ *p* < 0.001 between the untreated and LPS-treated (1 µg/mL) cells; * *p* < 0.05, ** *p* < 0.01, and *** *p* < 0.001 between the LPS (1 µg/mL) and MSLEO-treated LPS-induced cells.

In the present work, the effects of various concentrations of MSLEO on endogenous antioxidant enzyme (CAT, SOD, GPx, and GSH) activities were measured via ELISA using the LPS-induced RAW 264.7 cell model. The exposure of RAW 264.7 macrophage cells to 1 µg/mL LPS for 24 h resulted in a significant reduction in the antioxidant enzymes CAT, SOD, GPx, and GSH as compared to the untreated group (Figure 6). This reduction may be due to the innate immune response of macrophages to LPS, resulting in excessive production of ROS and RNS as a defense mechanism [117]. The long-term occurrence of excessive ROS and RNS or their inefficient scavenging impair the antioxidant enzymes’ ability to defend against damage from oxidative and nitrative stress [100], which in turn are linked to protein modifications, DNA damage, apoptosis, etc. [118,119]. 

However, when LPS-stimulated cells were co-cultured with varying dosages (12.5 and 100 µg/mL) of MSLEO, the levels of expression of CAT, SOD, GPx, and GSH were significantly elevated in a dose-independent manner. The antioxidant capacity of MSLEO could be due to the predominance of terpenes in the essential oil, which are considered natural antioxidants [120,121].

Jena et al. [19] reported that the essential oil of *Neocinnamomum caudatum* could ameliorate oxidative stress by upregulating the activities of CAT, SOD, GPx, and GSH. Similarly, the treatment of porcine small intestinal epithelial (IPEC-J2) cells with different doses of Oregano (*Origanum vulgare) essential oil resulted in a substantial* increase in SOD and CAT mRNA expression levels, as well as intracellular concentrations of SOD and GPx [122]. Working on bleomycin-induced pulmonary fibrosis in a murine model, Tavares et al. [123] demonstrated that *Cymbopogon winterianus* essential oil can significantly reduce inflammation in Bronchoalveolar Lavage Fluid (BALF), reduce malondialdehyde (MDA) levels, and increase SOD activity. The findings of this present experiment also revealed that MSLEO has the ability to reinstate the expression levels of the endogenous antioxidant enzymes by significantly increasing the production levels of CAT, SOD, GPx, and GSH compared to the LPS-stimulated group, which is essential for preventing many inflammation-related disorders [124]. 

### 2.8. MSLEO Prevents Mitochondrial Membrane Potential (Δψm) from LPS-Stimulated Depolarization

The mitochondrial membrane potential (ΔΨm) is the most important component in the energy storage process during oxidative phosphorylation and the maintenance of mitochondrial homeostasis through mitochondrial removal by autophagy [125]. Therefore, cell health can be monitored from the mitochondrial membrane potential. The expression of key tricarboxylic acid (TCA)-cycle-related enzymes and mitochondrial membrane potential decreases when LPS is activated, whereas the levels of microtubule-associated protein LC3b and ROS increase [126]. The effect of MSLEO on mitochondrial damage and mitochondrial membrane potential (ΔΨm) resulting from LPS-induced inflammation were assessed using the JC-1 (5,5,6,6’-tetrachloro-1,1’,3,3’ tetraethylbenzimi-dazoylcarbocyanine iodide) Mitochondrial Membrane Potential (MMP) Assay Kit. JC-1 emitted red fluorescence when the membrane potential was normal, but in the event of disruption, it emitted green fluorescence.

**Figure 7:**
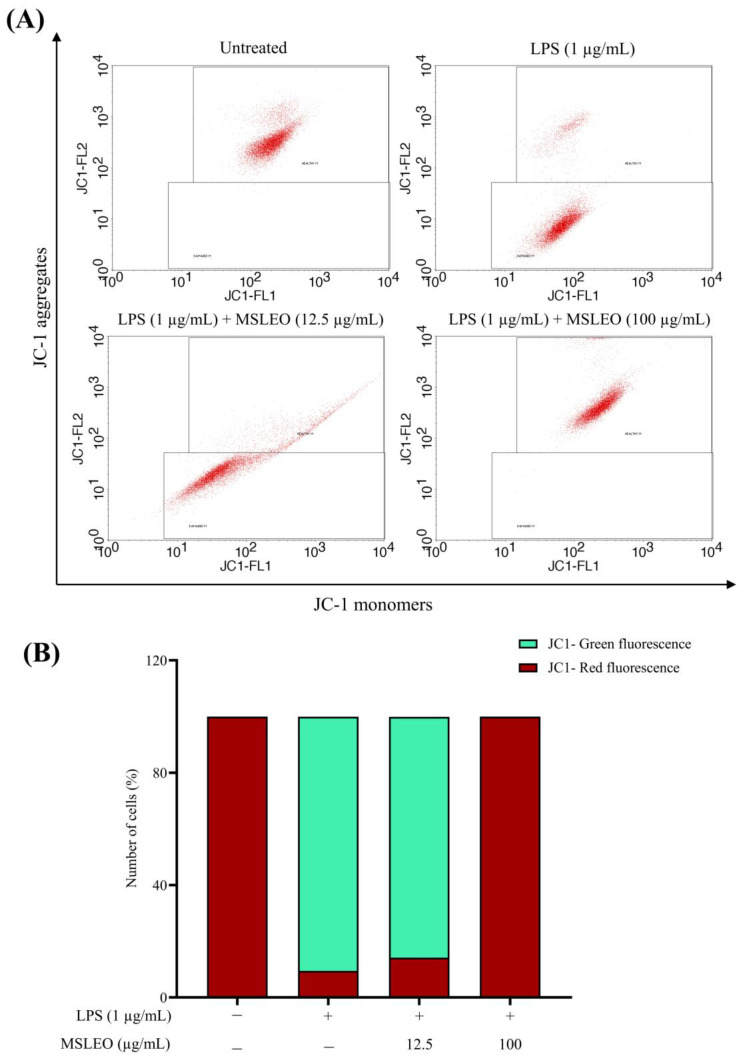
Effect of MSLEO on mitochondrial membrane potential (△ψm). RAW 264.7 cells activated by LPS (1 µg/mL) were treated with 12.5 µg/mL and 100 µg/mL MSLEO for 24 h. Following incubation, cells were stained with JC-1 dye, and fluorescence intensity was measured using a flow cytometer. (**A**) Representative flow cytometry dot plots of JC-1 staining in different treatment groups. (**B**) Quantification of mitochondrial membrane potential by comparing the percentage of gated cells emitting red fluorescence and green fluorescence in different treatment groups. Each value indicates the mean ± SD of three independent experiments. Statistical significance was determined by one-way ANOVA followed by the Tukey test.

When macrophage cells were subjected to LPS (1 µg/mL) for 24 h, a substantial decrease in gated cell populations emitting red fluorescence (9.46%) was observed compared to untreated cells (99.93%), indicating a lower membrane potential (Figure 7). This is because the aggregated form of JC-1 leaked out from the mitochondria into the cytoplasm as J-monomers, resulting in a decline in red fluorescence and an increase in green fluorescence, implying mitochondrial membrane dysfunction and depolarized MMP. Incubating these LPS-only-treated cells with MSLEO (12.5 and 100 µg/mL) for 24 h increased the level of red fluorescence. MSLEO, at 100 µg/mL, was also able to significantly increase the fraction of cells emitting red fluorescence up to 99.93%, pointing to higher mitochondrial membrane potential. This red spectral shift is due to an increased concentration of J-aggregates in mitochondria, suggesting the anti-inflammatory properties of *M*. *suaveolens* essential oil, which could regain the loss of mitochondrial membrane potential (Δψm) caused by LPS stimulation, depending on the concentration used. *Neocinnamomum caudatum* essential oil at concentrations of 12.5 and 25 µg/mL could significantly restore the mitochondrial membrane integrity [19]. de Carvalho et al. [127] found that the essential oil of *Eugenia uniflora* promoted mitochondrial membrane dysfunction in *Drosophila melanogaster* by inhibiting oxidative phosphorylation. Similarly, essential oils isolated from several species of *Cistus* are reported to ameliorate ultraviolet-B-induced mitochondrial damage and cellular senescence in human keratinocytes [128]. Essential oils containing β-caryophyllene as a major constituent in plant species, such as *Pterodon emarginatus, Aloysia citrodora, Syzygium aromaticum, Eplingiella fruticosa, Ocimum basilicum*, and *Salvia rosmarinus*, have the potential to counter oxidative stress and mitochondrial dysfunction and have possible links to neuroprotection [129].

### 2.9. MSLEO Restricts NF-κB Nuclear Translocation in LPS-Stimulated RAW 264.7 Cells

NF-κB, a heterodimer complex consisting of p65 and p50 subunits, regulates multiple innate immune functions, in addition to contributing to inflammation, cancer, and nervous system function [130]. Once activated, NF-κB regulates the expression of several related genes and facilitates the over-production of proinflammatory mediators, thereby promoting cell growth, proliferation, apoptosis, and disease progression [131,132,133,134,135]. In view of this, understanding the mechanism of the regulation of the NF-κB pathway is essential to study the pathogenesis and treatment of several human diseases.

**Figure 8:**
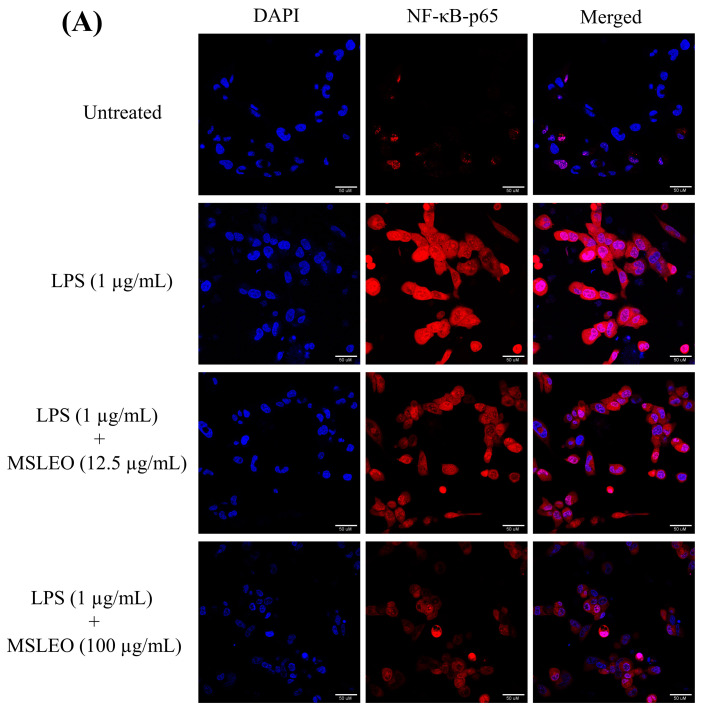

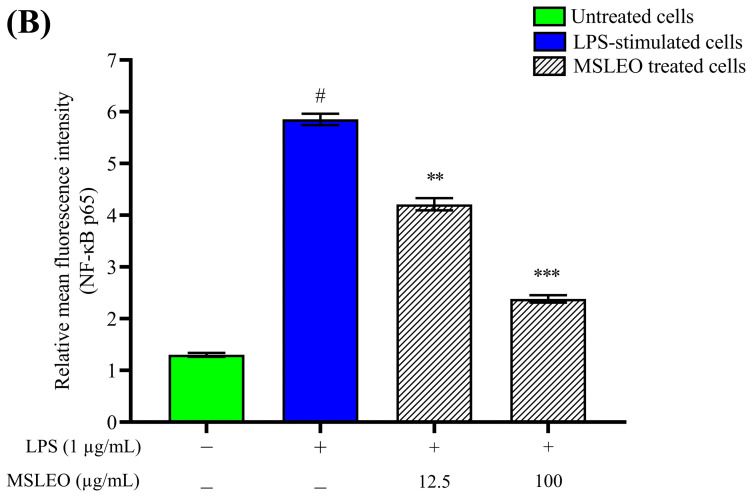
Effect of MSLEO on NF-κB-p65 translocation. (**A**) Pre-LPS-stimulated cells were incubated with different concentrations (i.e., 12.5 µg/mL and 100 µg/mL) of MSLEO for 24 h. After incubation, cells were stained with PE mouse anti-NF-κB p65 antibody conjugated with phycoerythrin fluorochrome (PE), and nuclei were counterstained with DAPI and incubated for 10 min. Cells were visualized under a confocal microscope (scale bar = 100 µM) to detect the localization of NF-κB-p65 by immunofluorescence analysis. (**B**) Bar graph representing the relative mean fluorescence intensity of NF-κB-p65 in untreated, LPS-alone-treated, and LPS + MSLEO-treated RAW 264.7 cells. Each value indicates the mean ± SD of three independent experiments. Statistical significance was calculated by one-way ANOVA followed by the Tukey test. ^#^ *p* < 0.001 between the untreated and LPS-treated (1 µg/mL) cells; ** *p* < 0.01 and *** *p* < 0.001 between the LPS (1 µg/mL) and MSLEO-treated LPS-induced cells.

In the current work, murine macrophage cells were exposed to MSLEO for 24 h, followed by LPS treatment. The inhibitory effect of MSLEO on the NF-κB signaling pathway was evaluated by measuring the NF-κB-p65 fluorescence intensity using phycoerythrin (PE) fluorochrome. In order to observe the translocation of NF-κB p65 from the matrix of the cytoplasm to the nucleus, confocal microscopy was used. The cells were then immuno-stained with the p65 antibody and incubated for 12 h. The nucleus was stained with a Hoechst 33342 fluorescent solution and an FITC-conjugated secondary antibody. As illustrated in Figure 8, LPS (1 μg/mL) stimulation for 24 h triggered the translocation of NF-κB dimers to the nucleus, and further phosphorylation of p65 resulted in a substantial increase in the relative mean fluorescence intensity of NF-κB-p65 expression, increasing by 4.6-fold in comparison with the untreated cells. However, MSLEO treatment with 12.5 μg/mL and 100 μg/mL doses could nullify these changes by restricting the translocation of NF-κB dimers, thereby lowering the relative mean fluorescence intensity of NF-κB-p65 by 28.57% (1.4-fold) and 60% (2.5-fold), respectively. The results confirmed the anti-inflammatory potential of MSLEO in RAW 264.7 cells. 

The NF-κB protein, being a central inflammatory mediator, has a major role in the pathogenesis of a good number of inflammatory disorders [136]. The essential oils of *Eucalyptus* species and *Neocinnamomum caudatum* are reported to attenuate LPS-stimulated inflammation in macrophages by inhibiting the activation of the NF-κB signaling pathway [19,137]. In another study by Mohamed et al. [78], it was shown that *Achillea millefolium* essential oil is responsible for the downregulation of the expression of TNF-α and IL-6 through the inhibition of NF-κB expression and the upregulation of peroxisome proliferator-activated receptor gamma (PPAR-γ). The mechanism of the anti-inflammatory effects of essential oils in several plant species, such as *Blumea balsamifera*, *Thymus vulgaris, Chamaecyparis obtusa*, *Citrus aurantium* var. *amara*, *Zanthoxylum bungeanum*, *Abies holophylla*, *Perilla frutescens*, *Mentha pulegium*, *Eucalyptus* spp., *Mentha arvensis*, *Glechoma hederaceai,* and *Zingiber striolatum*, has been found to be through the attenuation of the NF-κB signaling pathway [12]. 

The findings of the present study demonstrate that MSLEO exerts significant anti-inflammatory action by preventing the activation of the NF-κB pathway, which, in turn, inhibits the expression of proinflammatory cytokines, such as IL-1β, IL-6, and TNF-α; other inflammatory mediators, like iNOS and COX-2; and the regulation of the transcription of many genes, thereby regulating cell growth, differentiation, and apoptosis.

## 3. Materials and Methods

### 3.1. Collection of Plant Samples and Essential Oil Isolation

Fresh leaf samples of *Mesosphaerum suaveolens* were collected from plants in the flowering stage (Figure S2) from Balugaon, Chilika (85°15′54.2808″ E, 19°48′47.7144″ N; Altitude 17 m) of Khordha district, Odisha, India, in October 2021. The plant was identified by Prof. P. C. Panda, Taxonomist, and a voucher specimen (2417/CBT/Dt 13.10.2021) was housed in the herbaria of the Centre for Biotechnology, Siksha ‘O’ Anusandhan (Deemed to be University), Bhubaneswar, Odisha, India. The leaves were cut into small pieces, dried in the shade for 10 days, and made into fine powder. Then, 300 g of leaf powder was hydro-distilled in a Clevenger apparatus for 6 h. The isolated essential oil (MSLEO) was dried in anhydrous sodium sulfate (Na_2_SO_4_) and stored at 4 °C for subsequent analyses. 

### 3.2. Culture and Maintenance of Cells

RAW 264.7 murine macrophage cells were procured from the National Centre for Cell Science (NCCS) Cell Repository, Pune, Maharashtra, India. The cells were cultured in Dulbecco’s Modified Eagle Medium (DMEM), high-glucose medium supplemented with 10% FBS, 1% antibiotic–antimycotic solution, and 1% L-glutamine (200 mM) and incubated in a humidified CO_2_ incubator with an environment of 18–20% O_2,_ 5% CO_2_, and 37 °C. The cells were passaged every 2 days and allowed to proliferate for 24 h before the treatments.

### 3.3. GC-MS and GC-FID Analysis of MSLEO

The phytochemical composition of MSLEO was determined using a Clarus 580 Gas Chromatograph (Perkin-Elmer, Shelton, Connecticut, USA) coupled with an SQ8S MS detector. The analysis was conducted on an Elite-5 MS capillary column with an electron ionization source set at 70 eV. Helium was used as a carrier gas by maintaining the flow rate at 1 mL/min. A total of 0.1 µL of the undiluted essential oil was injected for GC-MS analysis. Initially, the oven temperature was set at 60 °C, which was gradually increased to 220 °C at a rate of 3 °C/min and held for 7 min. The injector temperature was set at 250 °C, and the transfer interface temperature was kept at 150 °C. The chemical compounds were identified by comparing the mass spectra of the detected compounds with the in-build NIST Mass Spectral library and by matching the retention indices (RIs) obtained by running straight-chain n-alkane series (C_8_–C_20_) under identical conditions with the published bibliographic literature [57].

Furthermore, to reinforce the reliability of the phytochemical identification of MSLEO, analysis was performed using the GC equipped with a Flame Ionization Detector (FID) while maintaining identical column conditions and programming parameters to those in the GC-MS analysis. To determine the response factors (RFs) of individual compounds, quantitative analysis was conducted with nonane, which served as the internal standard. The area percentages obtained from the GC-FID analysis were corrected using the response factors, which were established using representative reference standards for each group of chemicals under similar experimental conditions. The representative standard compounds used in the present study were as follows: eucalyptol (ether), 1-octen-3-ol (fatty alcohol), terpinen-4-ol (monoterpene alcohol), sabinene (monoterpene hydrocarbon), fenchone (monoterpene ketone), β-caryophyllene (sesquiterpene hydrocarbon), spathulenol (sesquiterpene alcohol), caryophyllene oxide (sesquiterpene oxides), hexahydrofarnesyl acetone (sesquiterpene ketone), abietatriene (diterpene hydrocarbons), phytol (diterpene alcohols), and dehydroabietol (oxygenated diterpenes). The response factor (RF) was calculated by using the formula:

RF = C_analyte_/[(A_analyte_/A_istd_)] × C_istd_.

where A_analyte_ and C_analyte_ are the GC peak area and the corresponding concentration of the standard compound representing a chemical group, and A_istd_ and C_istd_ are the GC peak area and concentration of the internal standard.

### 3.4. Cytotoxicity Assay

The MTT assay is a well-established quantitative colorimetric assay based on the conversion of MTT [3-(4,5-dimethylthiazol-2-yl)-2,5 diphenyl tetrazolium bromide] to an insoluble formazan crystal by succinate dehydrogenase to determine the toxicity level of a test compound in cultured cells [138]. In the present study, RAW 264.7 cells at a density of 2 × 10^5^ cells/mL were seeded in a 96-well plate, and the cells were allowed to proliferate and adhere properly for 24 h prior to treatment with the test compounds. The macrophage cells were then exposed to various doses of the essential oil (diluted with DMSO), MSLEO (12.5, 25, 50, and 100 µg/mL), and incubated at 37 °C for an additional 24 h in a 5% CO_2_ environment. After 24 h of incubation, the utilized medium was removed, and MTT reagent (0.5 mg/mL) was added to each well and further incubated for another 3 h. The medium was again taken out, 100 µL of DMSO was added to each well, and the plate was gently agitated until the complete dissolution of formazan crystals. Subsequently, the absorbance of the purple-colored formazan was measured with an ELISA plate reader at 570 nm. The cytotoxicity level of MSLEO was determined by comparing the absorbance of the MSLEO-treated cells with the absorbance of the untreated RAW 264.7 cells. The morphological alterations in RAW 264.7 cells after treatment with MSLEO were visualized under an inverted microscope (scale bar = 100 µm). The proportion of viable cells was determined by using the following mathematical expression:

$$Cell\;viability\left(\%\right)=\left(\frac{Absorbance\;of\;the\;treated\;cells}{Absorbance\;of\;the\;untreated\;cells}\right)\times 100$$

### 3.5. Annexin V-FITC/PI Apoptosis Assay

Annexin V-FITC/PI staining is one of the most extensively used tests to evaluate apoptosis and necrosis in cells [139,140]. In the current experiment, the percentage of apoptotic/necrotic RAW 264.7 cells were determined following the manual instructions provided with the Annexin V-FITC apoptosis kit (BD Biosciences). The cells were plated at a density of 2.5 × 10^5^ cells/mL in a 24-well plate and cultured for 24 h at 37 °C in a CO_2_ incubator. Then, the spent medium was aspirated out, and the cells were stimulated with LPS (1 µg/mL) for 2 h, except for the untreated cells. Subsequently, the stimulated cells were exposed to the minimum (12.5 µg/mL) and maximum (100 µg/mL) doses of MSLEO and incubated for another 24 h under similar conditions to those described earlier. After that, the cells were washed twice with 1X phosphate-buffered saline (PBS) solution, harvested, and resuspended in polystyrene tubes containing 400 μL of binding buffer supplemented with 5 μL of Annexin V-FITC and 5 μL of propidium iodide (PI). Fluorescence-activated cell sorting (FACs) tubes were kept in the dark at room temperature for 15 min, and then the cells were analyzed with a flow cytometer at an excitation wavelength of 488 nm and emission wavelengths of 530 nm and 610 nm for Annexin V-FITC and PI fluorescence, respectively. The anti-apoptotic potential of MSEO was assessed based on the proportion of viable (Annexin V^−ve^/PI^−ve^), early apoptotic (Annexin V^+ve^/PI^−ve^), and late apoptotic/necrotic (Annexin V^+ve^/PI^+ve^) cells screened using BD CellQuest Pro Ver. 6.0 software (Becton, Dickinson, Heidelberg, Germany). 

### 3.6. Proinflammatory Cytokine (IL-6, IL-1β, and TNF-α) Detection by ELISA

The levels of the expression of proinflammatory cytokines were evaluated using commercially available mouse immunoassay ELISA kits and referring to the manufacturer’s instructions (Abcam Co. Cambridge, UK). Initially, the cells were pretreated with LPS (1 μg/mL) for 2 h (except for the untreated group), and then these cells were subjected to lower and higher concentrations of MSLEO (12.5 and 100 µg/mL) for 24 h at 37 °C in the incubator. After incubation for 24 h, the culture medium was collected and centrifuged at 2000 rpm for 10 min at 4 °C. Afterward, supernatants were collected, and the absorbance was recorded at 450 nm to measure the levels of IL-6, IL-1β, and TNF-α. The results were expressed in ng/mL. 

### 3.7. RNA Extraction and Real-Time Quantitative PCR (RT-qPCR)

RT-qPCR analysis was conducted to estimate the levels of the expression of iNOS and COX-2 mRNA in LPS-stimulated cells following the protocol of Syam et al. [141]. As described above, the cells were treated with two doses of MSLEO, i.e., 12.5 µg/mL and 100 µg/mL, and incubated for 24 h at 37 °C and 5% CO_2_. After incubation, total RNA was isolated from the cells using the Qiagen RNeasy kit (Hilden, Germany) following the instructions provided by the manufacturer. The isolated RNA was treated with DNAse to avoid any genomic contamination. The purity of RNA was checked spectrophotometrically by comparing the absorbances taken at 260 nm and 280 nm, and the total RNA was quantified with the help of QiaExpert (Hilden, Germany). Later, the IScript cDNA synthesis kit (Bio-Rad) was used for the synthesis of cDNA from the previously isolated RNA through reverse transcription. Then, PCR amplification of the target genes, i.e., COX-2, iNOS, and GADPH (housekeeping gene), was performed by employing the cDNA as a template and gene-specific forward and reverse primers (Table 2). The RT-qPCR analysis was carried out with a Qiagen Rotor-Gene Q 5plex HRM RT-qPCR using SYBR green fluorescent dye with a thermal cycle of initial denaturation at 95 °C for 5 min, followed by 40 cycles of denaturation at 95 °C for 10 s, annealing at 60 °C for 20 s, and extension at 72 °C for 20 s. The 2^−ΔΔCT^ approach was used to measure and normalize the target genes’ expression levels, i.e., COX-2 and iNOS, relative to the internal housekeeping gene (GADPH) in the same sample.

### 3.8. Intracellular ROS Assay

D_2_CFH-DA (2,7-dichlorofluorescein diacetate) is a ROS-sensitive fluorescence indicator widely used for measuring intracellular ROS levels [142]. In viable cells, esterase cleaves DCFH-DA into DCFH, which is then oxidized by ROS to produce the highly fluorescent chemical DCF (2′,7′-dichlorofluorescein). Briefly, the cells were seeded at a density of 2.5 × 10^5^ cells/mL in a 24-well plate, pre-stimulated with LPS (1 μg/mL), and exposed to two concentrations of MSLEO (12.5 and 100 µg/mL) in 5% CO_2_ at 37 °C for 24 h. Subsequently, the used medium was taken out, and the cells were washed with 1X PBS and incubated for another 30 min with DCFH-DA (10 μM) away from light. DCFH-DA was then removed, and the cells were rinsed twice with cold PBS, trypsinized, and centrifuged. The collected pellets were then dissolved in PBS, and fluorescence intensity was measured using a flow cytometer (BD FACScan, BD Bioscience, San Jose, CA, USA).

### 3.9. Measurement of Endogenous Antioxidant Enzyme Activities by ELISA

Like in the endogenous antioxidant enzyme expression study, pre-stimulated cells grown to a density of 2 × 10^5^ cells/mL in a 24-well plate were subjected to two different doses (i.e., 12.5 µg/mL and 100 µg/mL) of MSLEO for 24 h at 37 °C and 5% CO_2_. The incubated cells were then washed with PBS, and the cellular lysates were collected using RIPA buffer and kept at −80 °C for further analysis. Following the manufacturer’s protocol, the activities of the endogenous antioxidant enzymes CAT, SOD, GPx, and GSH were measured spectrophotometrically using the cellular lysates. The activities of the endogenous antioxidant enzymes (CAT, SOD, GPx, and GSH) were expressed as mU/mL, U/mg, ng/mL, and µM protein, respectively.

### 3.10. Mitochondrial Membrane Potential (MMP) Assay 

The mitochondrial membrane potential (△ψm) was evaluated using JC-1 dye as per the protocol described by Yuan et al. [143]. The cells (2.5 × 10^5^ cells/mL) were seeded in a 24-well plate overnight after being treated with 1 μg/mL LPS for 2 h. They were then treated with 12.5 µg/mL and 100 µg/mL MSLEO and incubated at 37 °C and 5% CO_2_ for 24 h. The cells were subsequently washed with 1X PBS, trypsinized, and centrifuged, and following that, 0.5 mL of JC-1 working solution was added to the collected pellets. After that procedure, the cells were once again rinsed with PBS, resuspended in 1 mL of fresh 1X PBS, and then examined using a flow cytometer (Becton Dickinson FACScan, BD Bioscience, USA). The mitochondrial membrane potential was estimated by comparing the proportion of gated cells emitting red fluorescence (JC-1 aggregates) and green fluorescence (JC-1 monomers) measured at 540/570 nm and 485/535 nm, respectively. 

### 3.11. NF-κB Nuclear Translocation Assay by Confocal Microscopy

The cells were plated at a density of 2.5 × 10^5^ cells/mL in a 24-well plate and stimulated with LPS (1 μg/mL) for 2 h (except for the untreated ones). The pre-stimulated cells were then treated with 12.5 µg/mL and 100 µg/mL MSLEO and incubated for an additional time of 24 h at 37 °C in a 5% CO_2_ environment. The culture medium was subsequently taken out from each well, and cells were rinsed twice with 1X PBS; the cells grown in the wells were fixed using 0.5 mL of BD Cytofix/Cytoperm solution (BD Biosciences, 554714) at room temperature for 10 min. After washing the cells with 1X PBS (supplemented with 0.5% bovine and 0.1% sodium azide), the cells were incubated for 30 min with mouse anti-NF-κB p65 antibody (10 µL) conjugated with phycoerythrin fluorochrome (PE). For nuclear staining, cells were counterstained with 100 µL of DAPI solution (1 µg/ mL) and incubated for 10 min in the dark. After the final wash, the cells were visualized under a Zeiss LSM 880 (Carl Zeiss Microscopy GmbH, Jena, Germany) confocal microscope, and the expression level of NF-κB p65 was measured using Image J software version 1.54F. 

### 3.12. Statistical Analysis

All the findings from the experiments are reflected as mean ± SD (standard deviation). GraphPad Prism version 8.0 software (Boston, MA, USA) was used to evaluate the statistical significance by ANOVA test, followed by Tukey’s multiple range test.

## 4. Conclusions

The present research unraveled the anti-inflammatory potential of *Mesosphaerum suaveolens* leaf essential oil (MSLEO) and the molecular mechanism of its action for the first time. The study revealed that MSLEO could inhibit LPS-induced inflammation and oxidative stress in RAW 264.7 macrophages through a reduction in the levels of the proinflammatory cytokines like IL-6, IL-1β, and TNF-α, the expression of inflammatory mediators such as iNOS and COX-2, and intracellular ROS production. MSLEO was also able to maintain the mitochondrial membrane integrity and inhibited the translocation of NF-κB from the cytosol to the nucleus, thereby downregulating the expression of proinflammatory genes, cytokines, and mediators. The findings of the current research indicate that MSLEO could serve as a promising therapeutic agent against inflammation-related responses. However, further in-depth laboratory-based research involving in vitro assays and in vivo models may be necessary to establish the therapeutic efficacy of the essential oil before initiating preclinical trials on its anti-inflammatory effects, leading to drug development.

## Figures and Tables

**Table 1:** Chemical composition of *Mesosphaerum suaveolens* leaf essential oil (MSLEO).

S. No.	Compound ^a^	Ri ^b^	Ri ^c^	Peak Area %	RF ^d^
1	Sabinene	966	975	0.21 ± 0.01	1.0
2	1-Octen-3-ol	973	979	0.34 ± 0.01	1.5
3	Eucalyptol	1028	1031	1.70 ± 0.04	1.3
4	Fenchone	1083	1086	0.14 ± 0.01	1.3
5	Terpinen-4-ol	1175	1177	0.76 ± 0.02	1.3
6	*α*-Cubebene	1337	1351	0.17 ± 0.01	1.0
7	*α*-Copaene	1367	1376	0.87 ± 0.03	1.0
8	*β*-Elemene	1380	1390	0.41 ± 0.02	1.0
9	*α*-Gurjunene	1396	1409	0.16 ± 0.01	1.0
10	*β*-Caryophyllene	1418	1419	16.17 ± 0.62	1.0
11	trans*-α*-Bergamotene	1426	1434	1.82 ± 0.07	1.0
12	*α*-Humulene	1446	1454	1.40 ± 0.05	1.0
13	allo-Aromadendrene	1450	1460	0.28 ± 0.01	1.0
14	*β*-Chamigrene	1462	1477	0.15 ± 0.01	1.0
15	*γ*-Muurolene	1470	1479	0.51 ± 0.02	1.0
16	*β*-Selinene	1479	1490	1.25 ± 0.03	1.0
17	Viridiflorene	1481	1496	0.17 ± 0.01	1.0
18	*α*-Selinene	1487	1498	1.86 ± 0.04	1.0
19	*δ*-Cadinene	1508	1523	0.54 ± 0.02	1.0
20	Isocaryophyllene oxide	1539	1527	0.20 ± 0.01	1.5
21	Spathulenol	1575	1578	7.89 ± 0.28	1.3
22	Caryophyllene oxide	1577	1583	2.04 ± 0.08	1.5
23	Viridiflorol	1584	1592	0.23 ± 0.01	1.3
24	Humulene epoxide II	1598	1608	0.46 ± 0.01	1.5
25	10-epi-γ-Eudesmol	1606	1623	0.38 ± 0.02	1.3
26	*β*-Atlantol	1623	1608	0.46 ± 0.02	1.3
27	Caryophylla-4(12),8(13)-dien-5β-ol	1627	1640	0.24 ± 0.01	1.3
28	Selin-11-en-4-α-ol	1649	1659	2.10 ± 0.07	1.3
29	14-Hydroxy-9-epi-(E)-caryophyllene	1662	1669	1.02 ± 0.03	1.3
30	(Z)-α-trans-Bergamotol	1681	1690	1.46 ± 0.05	1.3
31	Hexahydrofarnesyl acetone	1829	1836	0.42 ± 0.02	1.3
32	Rimuene	1894	1896	0.52 ± 0.02	1.4
33	(3Z)-Cembrene A	1935	1966	0.18 ± 0.01	1.4
34	Manoyl oxide	1976	1977	1.90 ± 0.06	1.4
35	Phyllocladene	2004	2017	11.85 ± 0.43	1.4
36	Abieta-8,12-diene	2015	2022	0.16 ± 0.01	1.4
37	Abietatriene	2054	2056	11.46 ± 0.52	1.4
38	Kaur-16-ene	2059	2061	0.46 ± 0.03	1.4
39	Abietadiene	2085	2087	3.79 ± 0.18	1.4
40	Phytol	2114	2114	1.70 ± 0.08	1.3
41	Abieta-8(14),13(15)-diene	2140	2154	0.41 ± 0.02	1.4
42	Isopimara-7,15-dien-3-one	2225	2227	0.61 ± 0.02	1.4
43	Dehydroabietal	2256	2275	0.19 ± 0.01	1.4
44	Isopimarol	2303	2305	0.34 ± 0.03	1.3
45	8,13-Abietadien-18-ol	2318	2324	6.65 ± 0.32	1.4
46	4-Epiabietol	2349	2344	0.43 ± 0.01	1.4
47	Dehydroabietol	2358	2368	3.58 ± 0.16	1.4
48	Abietol	2392	2401	0.57 ± 0.02	1.3
	Ether			1.70 ± 0.04	
	Fatty alcohol			0.34 ± 0.01	
	Monoterpene alcohol			0.76 ± 0.02	
	Monoterpene hydrocarbon			0.21 ± 0.01	
	Monoterpene ketone			0.14 ± 0.01	
	Sesquiterpene hydrocarbons			25.74 ± 0.54	
	Sesquiterpene alcohols			13.76 ± 0.29	
	Sesquiterpene oxides			2.70 ± 0.06	
	Oxygenated sesquiterpene			0.42 ± 0.02	
	Diterpene hydrocarbons			28.82 ± 0.87	
	Diterpene alcohols			2.62 ± 0.08	
	Oxygenated diterpenes			13.34 ± 0.38	
	Total identified			90.55 ± 1.89	

Data are represented as mean ± SD (*n* = 3). ^a^ Compounds are listed in order of elution on Elite-5 MS column. ^b^ RI experimentally calculated using straight-chain n-alkane series (C_8_–C_20_). ^c^ RI obtained from literature [57]. ^d^ FID response factor (RF).

**Table 2:** Sequences of primer sets used in RT-qPCR.

Target Gene		Primer Sequence
iNOS	Forward	5′-CTTCAACACCAAGGTTGTCTGCA-3′
	Reverse	5′-ATGTCATGAGCAAAGGCGCAGAA-3′
COX-2	Forward	5′-CACTACATCCTGACCCACTT-3′
	Reverse	5′-ATGCTCCTGCTTGAGTATGT-3′
GAPDH	Forward	5′-GCAAAGTGGAGATTGTTGCCATC-3′
	Reverse	5′-CATATTTCTCGTGGTTCACACCC-3′

## Data Availability

The data presented in this work are available in the article.

## Supplementary Materials

The following supporting information can be downloaded at: https://www.mdpi.com/article/10.3390/molecules28155817/s1, Figure S1: GC-MS chromatogram of the Mesosphaerum suaveolens leaf essential oil (MSLEO). Figure S2: Image of Mesosphaerum suaveolens plant.

## Sample Availability

Samples of the compounds are not available from the authors.
