# Supplementary material for: Mesosphaerum suaveolens Essential Oil Attenuates Inflammatory Response and Oxidative Stress in LPS-Stimulated RAW 264.7 Macrophages by Regulating NF-κB Signaling Pathway
Source: Molecules. 2023 Aug 2;28(15):5817. doi: 10.3390/molecules28155817 (PMC10420984; doi:10.3390/molecules28155817)
Supplement: Supplementary file 1 [file molecules-28-05817-s001.zip › molecules-2468052-supplementary.pdf]

-Supplementary Materials-

Mesosphaerum suaveolens essential oil attenuates inflammatory response and oxidative stress in LPS-stimulated RAW 264.7 macrophages by regulating NF-κB signaling pathway

Omprakash Mohanta, Asit Ray, Sudipta Jena, Ambika Sahoo, Soumya Swarup Panda, Prabhat Kumar Das, Sanghamitra Nayak and Pratap Chandra Panda*

Centre for Biotechnology, Siksha 'O' Anusandhan (Deemed to be University), Kalinga Nagar, Bhubaneswar-751003, India
* Correspondence: author Email: pcpanda2001@yahoo.co.in

Contents:

Figure S1. GC-MS chromatogram of the *Mesosphaerum suaveolens* leaf essential oil (MSLEO).

Figure S2. Image of *Mesosphaerum suaveolens* plant

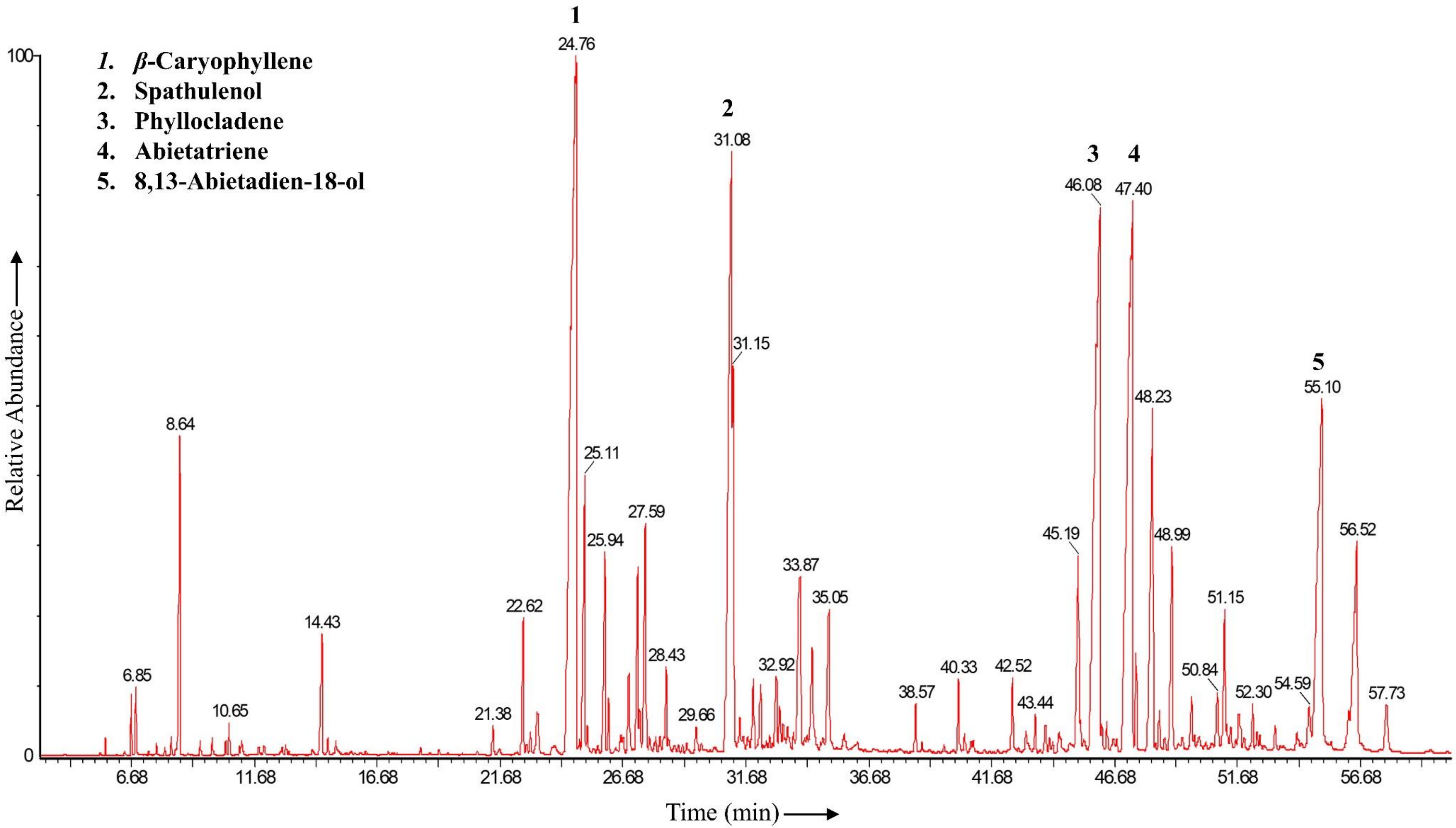

Figure S1. A representative GC-MS chromatogram of *Mesosphaerum suaveolens* leaf essential oil (MSLEO). Peaks corresponds to the five most dominant constituents, labeled as 1 to 5.

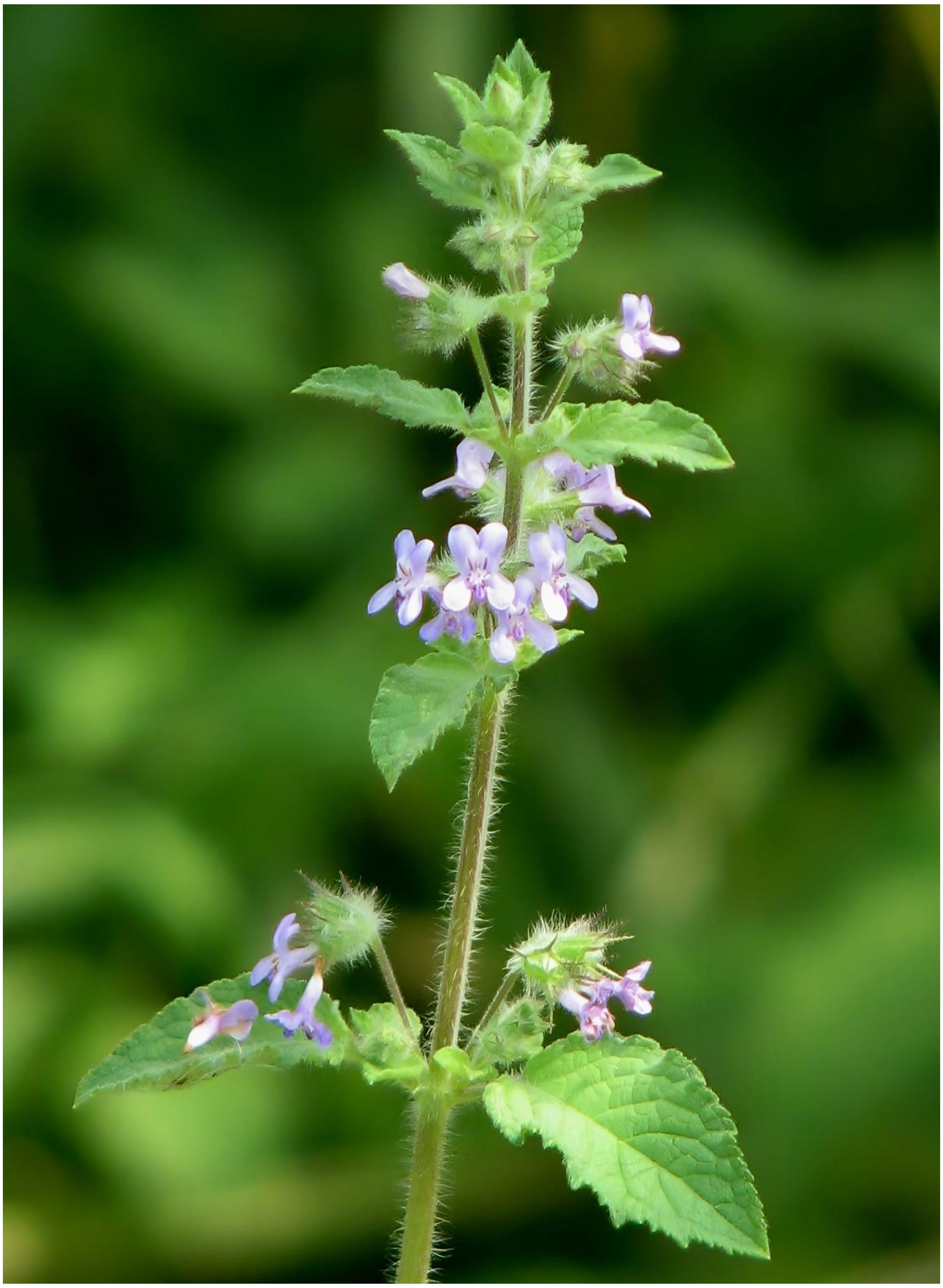

Figure S2. *Mesosphaerum suaveolens* plant
